# Hydrogen Embrittlement Behavior of 18Ni 300 Maraging Steel Produced by Selective Laser Melting

**DOI:** 10.3390/ma12152360

**Published:** 2019-07-25

**Authors:** Young Jin Kwon, Riccardo Casati, Mauro Coduri, Maurizio Vedani, Chong Soo Lee

**Affiliations:** 1 Graduate Institute of Ferrous Technology (GIFT), Pohang University of Science and Technology (POSTECH), Pohang 790-784, Korea; 2 Technical Research Laboratories, POSCO, Pohang 790-785, Korea; 3 Department of Mechanical Engineering, Politecnico di Milano, Via G. La Masa 34, 20156 Milan, Italy; 4 Department of Chemistry, University of Pavia, via Taramelli 12, 27100 Pavia, Italy

**Keywords:** maraging steel, hydrogen embrittlement, aging, selective laser melting, additive manufacturing

## Abstract

A study was performed to investigate the hydrogen embrittlement behavior of 18-Ni 300 maraging steel produced by selective laser melting and subjected to different heat treatment strategies. Hydrogen was pre-charged into the tensile samples by an electro-chemical method at the constant current density of 1 A m^−2^ and 50 A m^−2^ for 48 h at room temperature. Charged and uncharged specimens were subjected to tensile tests and the hydrogen concentration was eventually analysed using quadrupole mass spectroscopy. After tensile tests, uncharged maraging samples showed fracture surfaces with dimples. Conversely, in H-charged alloys, quasi-cleavage mode fractures occurred. A lower concentration of trapped hydrogen atoms and higher elongation at fracture were measured in the H-charged samples that were subjected to solution treatment prior to hydrogen charging, compared to the as-built counterparts. Isothermal aging treatment performed at 460 °C for 8 h before hydrogen charging increased the concentration of trapped hydrogen, giving rise to higher hydrogen embrittlement susceptibility.

## 1. Introduction

Maraging steels are low-carbon ultra-high strength alloys that are currently used for several applications such as engine components, high-wear parts of assembly lines, tools, and dies. Their high strength and toughness derive from the aging of a relatively soft Ni-rich martensite. In 18-Ni 300 alloy and similar grades, Ni-based precipitates, mainly Ni3Ti and Ni3Mo, form in a first stage of aging and are replaced in a second stage by Fe–Mo precipitates, namely Fe_2_Mo and Fe_7_Mo_6_ [1,2,3,4,5,6]. Prolonged aging treatment leads to austenite reversion that is stimulated by the release of Ni in the matrix due to the decomposition of the Ni_3_Ti and Ni_3_Mo phases [6,7,8,9].

Recently, maraging steels have attracted attention because they have revealed a capability to be successfully 3D-printed by laser-based technologies. Several works show their good processability by selective laser melting (SLM), which is the most widespread powder-bed additive manufacturing (AM) technology for metals. SLM is used for manufacturing structural components for mechanical, medical, and aerospace applications using different metals and alloys [10,11,12].

The study of the phenomena that may cause failure of AM components in service needs to be investigated in order to extend the application of these technologies to the production of structural parts. There is a strong need to fully understand the mechanisms underlying corrosion, fracture, fatigue, and creep behavior of 3D-printed metals in order to safely apply AM technologies in all those applications implying severe environmental conditions or heavy dynamic loads. In particular, the phenomena responsible for hydrogen embrittlement (HE) in steels produced by SLM has never been investigated to the authors’ knowledge, although it is of paramount importance, especially when high-strength steels are considered. Indeed, the interaction of a laser beam with high energy density over a small area of powder results in high heating and cooling rates of the metal and severe thermal gradients. The extreme processing conditions of SLM give rise to peculiar microstructures that may include metastable phases, supersaturated solid solutions, fine cellular structures, or crystallographic textures that, in turn, are responsible for the particular mechanical and functional properties of the SLM processed alloys [13,14,15].

This study aimed to investigate the HE behavior of an 18-Ni maraging steel (300 grade) produced by SLM. Extrinsic HE behavior of hydrogen-charged samples at the same current density was evaluated. Tests were conducted on materials subjected to different thermal histories before being hydrogen charged; i.e., the steel in the as-built state, after solution treatment, or after isothermal aging performed starting from both the as-built and the solution-treated conditions. Tensile tests were performed on H-charged and uncharged specimens. The fractured samples were heated to 600 °C to quantify the hydrogen desorption rate and concentration by mass spectroscopy.

## 2. Materials and Methods

Gas-atomized 18-Ni maraging steel (300 grade) powder with the chemical composition given in Table 1 was used for the experiments. A Renishaw AM250 SLM system, with a 200 W single mode fiber laser and an estimated beam diameter at the focal point of 75 µm, was used to produce a set of horizontal bars (10 mm × 10 mm × 75 mm). Block-type supports were built to join parts together with a carbon steel build plate. Laser melting was carried out under an Ar atmosphere by partially overlapped spots exposed to the radiation for 80 µs. The spot and hatch distance were set at 65 µm and 80 µm, respectively, whereas layer thickness was set at 40 µm. Laser scanning direction was rotated by 67° after each layer, in order to reduce formation of residual stresses and crystallographic textures. Aging treatment of the samples was performed at 460 °C for 8 h starting from the as-built condition, or after a standard solution treatment carried out at 815 °C for 30 min and followed by water quenching. The as-built and solution treated samples were named “AB” and ST”, respectively. The samples directly aged from the as-built state and those aged from the solution treated condition were named “AB-aged” and “ST-aged”, respectively. Microstructure analysis was carried out by optical microscope and scanning electron microscope (SEM) after chemical etching with 15 g CuCl_2_, 120 mL HCl, and 75 mL ethanol, and by transmission electron microscope (TEM).

**Figure 1:**
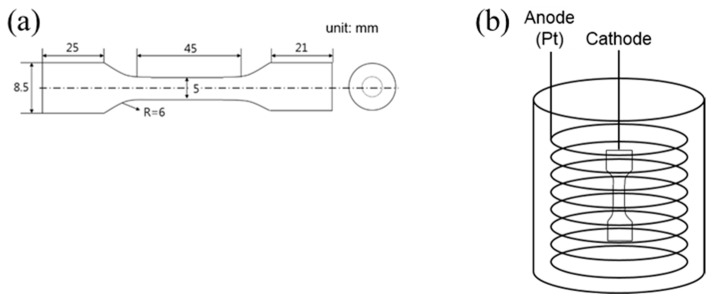
Schematic illustration of (**a**) tensile specimen and (**b**) electro-chemical H-charging cell.

The printed and heat-treated sample bars were machined in order to produce cylindrical dog-bone specimens with a gauge length of 45 mm and a diameter of 5 mm (Figure 1a). Tensile tests were performed at room temperature with an initial strain rate of 10–5 s^−1^, using universal testing machines (INSTRON 8501 or MTS Alliance RT/100) equipped with an extensometer. Fractured surfaces were observed by SEM. X-ray diffraction (XRD) patterns were collected using a Rigaku Smartlab instrument. Cu Kα (k = 0.15418 nm) radiation was employed. Peak identification and quantitative analysis of phases was performed by the Rietveld method.

Hydrogen was pre-charged into the tensile samples by the electro-chemical method. The samples were immersed in distilled water solution containing 3 wt.% NaCl and 0.3 wt.% NH_4_SCN and were then connected to a cathode. A Pt coil was connected to the anode (Figure 1b). Firstly, hydrogen ions in the solution were adsorbed by the sample surface and consequently absorbed. The process was performed at the constant current densities of 1 A m^−2^ and 50 A m^−2^ for 48 h at room temperature. After the tensile test, the desorption rate and concentration of hydrogen in broken dogbone specimens was quantified using quadrupole mass spectroscopy (Q-mass). The samples were heated from room temperature to 600 °C at a constant heating rate of 100 °C/h.

## 3. Results

**Figure 2:**
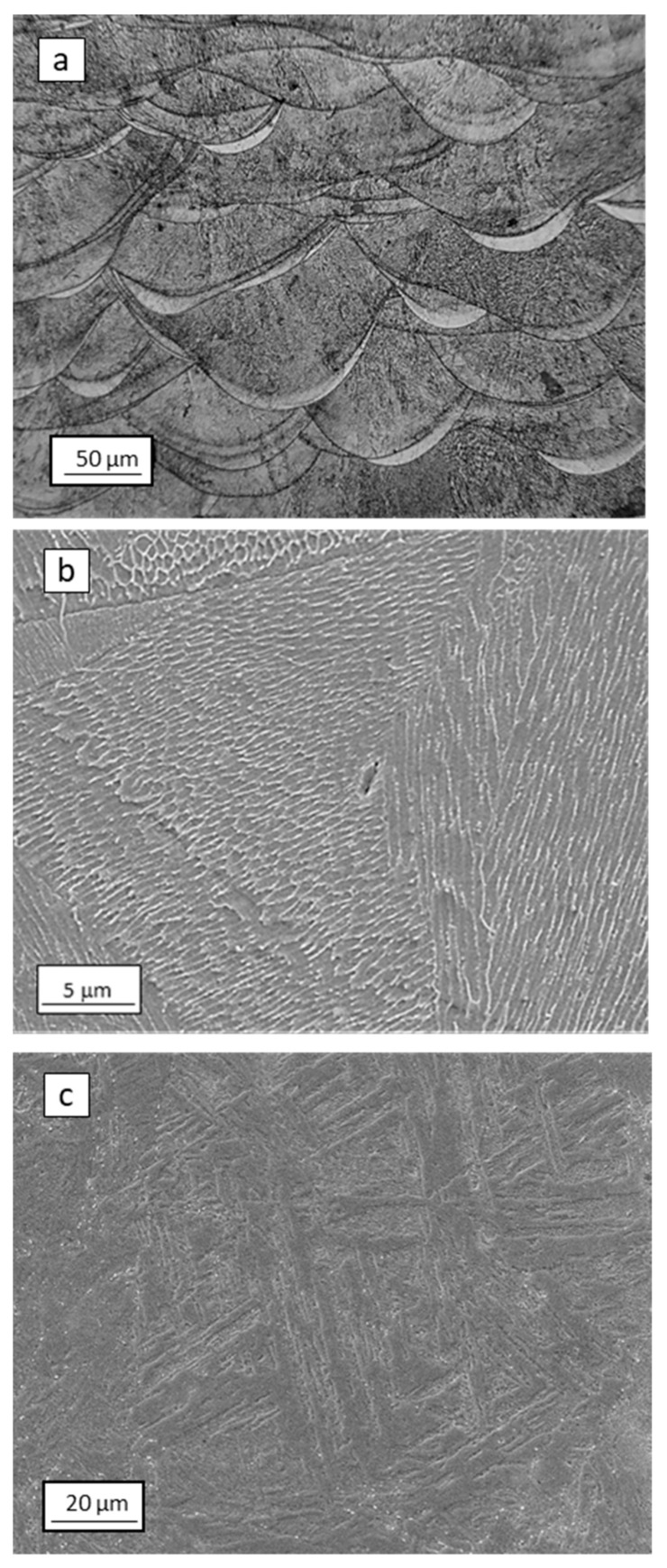
(**a**) Optical micrograph and (**b**) SEM image of as-built material; (**c**) SEM image of the alloy after solution treatment at 815 °C followed by water quenching.

**Figure 3:**
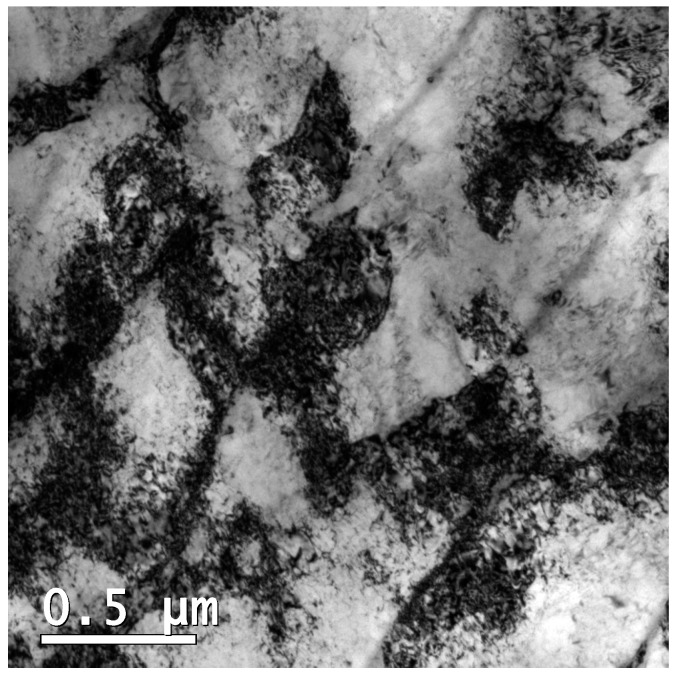
TEM image (bright field) of as-built material.

Maraging steel specimens were successfully produced by SLM and showed an average density of 99.8%. Figure 2a shows an optical micrograph of the section parallel to the building direction of the AB material. As already shown in previous works [6,12], solidified melt pool boundaries become visible after chemical etching. The high magnification SEM image of Figure 2b reveals the expected cellular solidification structure, characteristic of many highly-alloyed materials processed by SLM [6,12,13,14,15,16], with a cell size smaller than 1 μm. Cells are surrounded by a bright-contrast network that in previous works was identified as solute-rich high dislocation density regions [6,12,17]. Consistent with this, the high magnification bright field TEM image in Figure 3 shows high dislocation density regions surrounding the primary cells. After solution treatment performed at 815 °C for 30 min followed by water quenching, the solidification cellular structure was replaced by a martensitic structure, as depicted in Figure 2c. Rietveld analysis revealed that AB and AB-aged steels contained 11.4% and 15.6% of γ-iron, respectively, whereas ST and ST-aged steels contained 4.1% and 5.5% of γ-iron, respectively.

**Figure 4:**
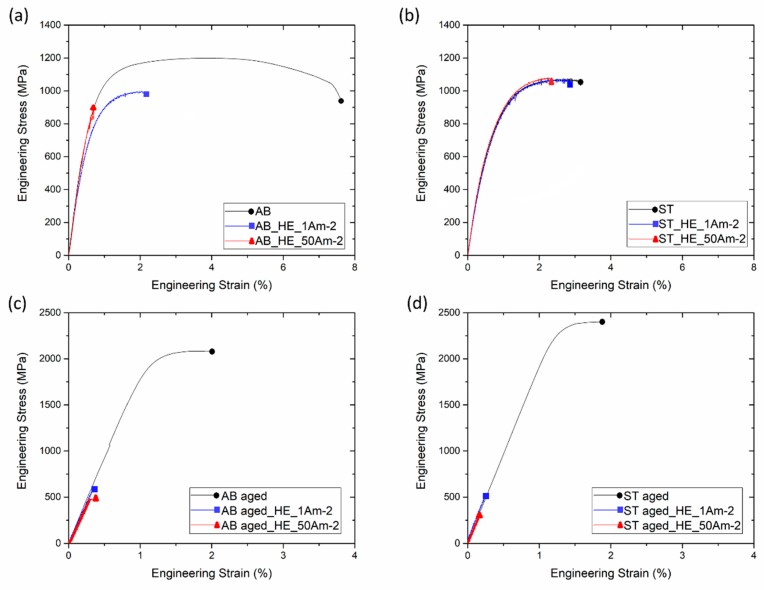
Stress-strain curves of (**a**) as-built (AB), (**b**) solution treated (ST), (**c**) AB-aged, and (**d**) ST-aged samples before and after H-charging at 1 A m^−2^ and 50 A m^−2^ for 48 h.

**Figure 5:**
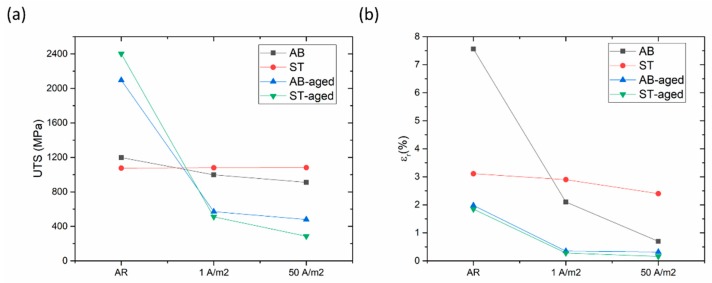
Overall trends of (**a**) ultimate tensile strength and (**b**) elongation at rupture as a function of current density levels applied for H-charging.

In Figure 4, the tensile curves of H-charged and uncharged specimens are reported. Tensile tests were performed on specimens that were doped with hydrogen using two different current density levels, namely 1 A m^−2^ and 50 A m^−2^. The trends of strength and elongation as a function of H-charging conditions are shown in Figure 5. AB alloy shows ultimate tensile strength (UTS) of 1178 MPa and elongation at rupture (ε_r_) of 7.6%. Solution treatment led to reductions in strength (UTS = 1076 MPa) and ductility (ε_r_ = 3.1%). Additional aging treatment for 8 h at 460 °C increased the strength of the AB alloy (UTS = 2095 MPa) and, in particular, that of the ST steel (UTS = 2401 MPa), but it also radically reduced the ductility of the materials. Indeed, the ε_r_ of AB-aged and ST-aged alloys was 2.0% and 1.9%, respectively.

Fractures occurred at lower strain values in H-charged specimens as compared to their uncharged counterparts. The premature fracturing was also responsible for the reduction of UTS. This tendency became more distinct in samples that underwent the most severe H-charging conditions (i.e., 50 A m^−2^ for 48 h). The sensitivity to HE varied depending on the thermal history of specimens. The degradation of tensile properties due to HE was found to be alleviated by solution treatment. Indeed, the ductility and strength of ST alloy remained almost unchanged even when samples underwent severe hydrogen charging at 50 A m^−2^ for 48 h (UTS = 1081 MPa; ε_r_ = 2.4%). Conversely, the loss in ductility became more severe after aging treatments, in samples aged from both the AB and the ST conditions.

**Figure 6:**
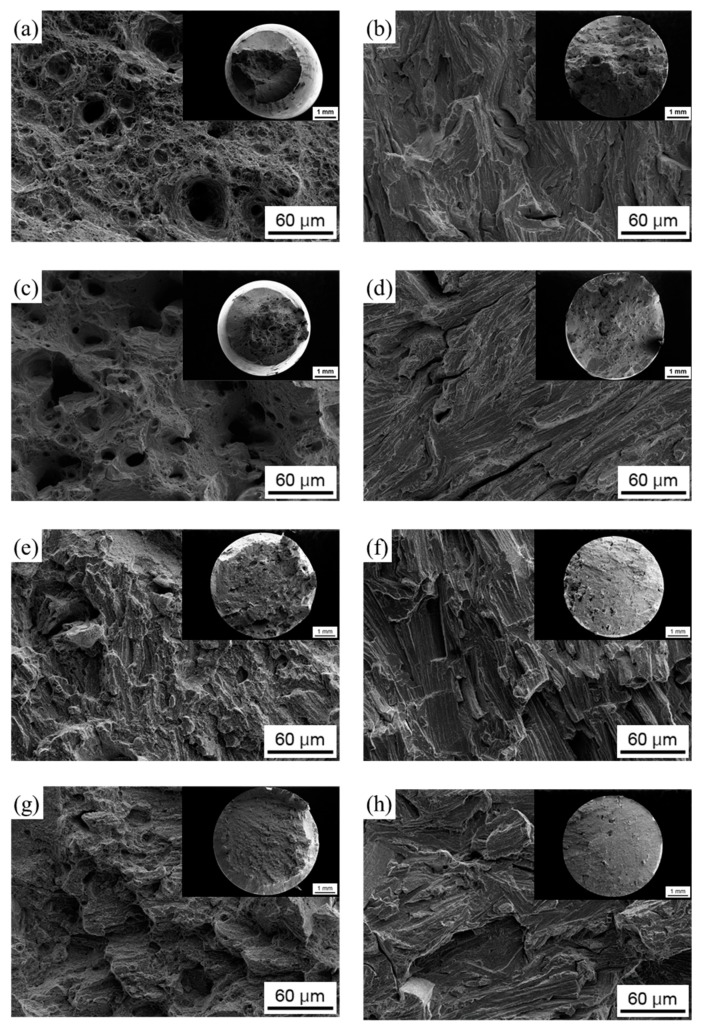
Fracture surfaces of (**a**,**b**) AB, (**c**,**d**) ST, (**e**,**f**) AB-aged, and (**g**,**h**) ST-aged samples; (**a**,**c**,**e**,**g**) before H-charging and (**b**,**d**,**f**,**h**) after H-charging.

SEM analysis was carried out to investigate the fracture modes of the maraging steel tested under different thermal conditions (Figure 6). AB and ST specimens showed local necking surrounding the fracture surfaces and, at higher magnification, typical fracture features of ductile materials, with dimples visible throughout the surface. H-charged AB and ST samples showed quasi-cleavage fracture surfaces with no evidence of dimples or necking. On fracture surfaces of AB- and ST-aged samples, even fewer ductile features were noticeable compared to the un-aged counterparts and evident cleavage planes appeared after H-charging.

**Figure 7:**
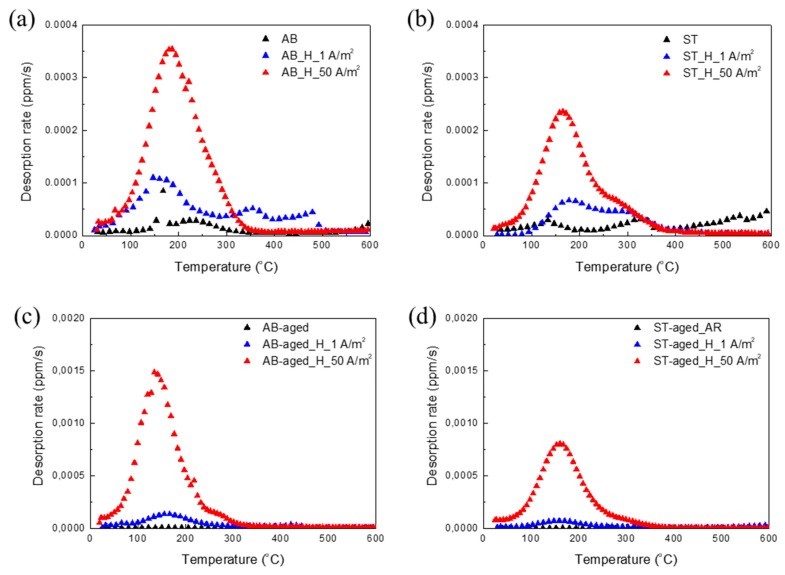
Thermal desorption curves for (**a**) AB, (**b**) ST, (**c**) AB-aged, and (**d**) ST-aged samples before and after H-charging at 1 A m^−2^ and 50 A m^−2^ for 48 h.

**Figure 8:**
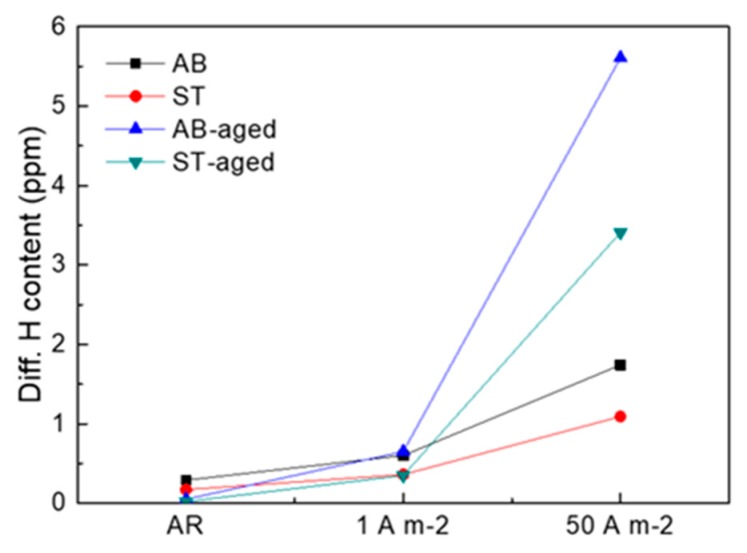
Overall trends of concentration of diffusible hydrogen as a function of H-charging conditions.

In Figure 7, the thermal desorption curves are reported for all the investigated materials and, in Figure 8, the estimated amounts of trapped hydrogen calculated as the integral of desorption rates are plotted as a function of the applied current densities. Regardless of thermal treatment or H-charging condition, the rate of hydrogen desorption gradually increased with temperature, reaching the maximum values between 150 °C and 200 °C. The total amount of trapped hydrogen evidently rises with increasing current density applied for H-charging. Furthermore, different thermal treatment routes influenced the hydrogen solubility of the steel. Indeed, the AB samples desorbed more hydrogen than ST samples and, in turn, AB-aged and ST-aged alloys released more hydrogen than their un-aged counterparts.

## 4. Discussion

This work focused on the mechanical behavior and HE susceptibility of maraging steel grade 300 produced by SLM and subjected to different thermal histories.

As shown in Figure 4 and Figure 5, steel samples that underwent solution annealing at 850 °C for 30 min showed lower tensile strength than the as-built alloy, likely because of the stress-relieving effect and slight microstructural coarsening. In ST samples, aging treatment performed at 460 °C for 8 h increased strength up to 2401 MPa, but reduced material ductility (εr decreased from 3.1% to 1.9%). The strengthening effect is ascribed to the formation of Ni_3_Ti and Ni_3_Mo precipitates during aging, as widely described in [1,2,3,4,5,6]. The same aging treatment performed on AB samples was responsible for an increase in UTS to 2095 MPa and for a drastic drop in εr from 7.6% to 2.0%. Previous works have showed that several alloys processed by SLM can be directly age-hardened without being previously solution-annealed and water quenched [12,18,19]. Indeed, the small melt pools generated by laser, which are a few tens of micrometers in width (Figure 2a), solidify and cool at a high rate, generating extended supersaturated solid solutions that can be effectively exploited by direct aging for precipitation strengthening of the alloy [12,18,19].

Hydrogen charging led to degradation of the tensile properties of maraging steel in all tested conditions. In AB alloy, strength and ductility reduced drastically after H-charging, especially in samples treated at 50 A m^−2^ for 48 h. In comparison, ST samples were less susceptible to H-charging. Indeed, the tensile properties of samples subjected to solution treatment remained almost constant after electro-chemical treatments performed at either 1 A m^−2^ or 50 A m^−2^. HE of steels is correlated with the attractive interactions between hydrogen atoms and structural defects. Crystal imperfections and defects such as vacancies, dislocations, interfaces, crack tips, or pores play a crucial role in the uptake, transport, and trapping of hydrogen atoms [20,21]. The solute hydrogen atoms diffuse to stress-concentration sites and lead to premature failures. Indeed, local accumulation of hydrogen atoms reduces bond strength between metal atoms, favoring crack initiation and propagation [20,22]. Thus, materials with fewer trapping sites for hydrogen typically present higher resistance to HE [23,24]. Solution treatment radically modified the as-built microstructure of the steel, including its microstructural constituents and concentration of hydrogen trapping sites, leading to a different HE behavior. Maraging samples produced by SLM showed a cellular solidification structure (Figure 2b). Consistent with previous works, we noticed that cell boundaries are regions with high dislocation density, where austenite grains are prone to form due to an increased content of γ-stabilizing elements [6,12,17,25]. Solution treatment at 815 °C for 30 min followed by water quenching replaced the cellular solidification structure surrounded by dislocations, typical of the as-built maraging steel processed by SLM, with lath martensite (Figure 2c) and drastically reduced the overall amount of retained austenite (from 11.4% to 4.1%). Hydrogen has higher solubility in FCC γ-Fe than in BCT α’-Fe [26] and dislocations, which act as trapping sites for hydrogen, further increase the amount of hydrogen that can be hosted by the steel [27]. For these reasons, AB samples released more hydrogen on heating compared to ST samples. Austenite is considered as less susceptible to HE compared to martensite or ferrite [26], and it has been shown that austenite has a positive effect on the HE behavior of maraging steels produced by conventional methods [28]. Nevertheless, the HE behavior of alloys with complex microstructures with two or more co-existing phases is difficult to predict. The worse HE behavior and, in particular, the drastic ductility drop showed by AB samples as compared to ST samples, can be attributed to the continuous dislocation network surrounding the solidification cells and to the high number of γ/α’ interfaces that can act as efficient trapping sites for hydrogen atoms and favorable nucleation sites for cracks [20,21,29]. In addition, in [30], the authors showed that retained austenite in steels can transform into strain-induced martensite on loading, leading to the diffusion of hydrogen atoms toward the interface between retained austenite and martensite laths. Thus, the local accumulation of hydrogen atoms at the interface could further contribute to premature cracking. The segregation of hydrogen at cell boundaries led to deterioration in the bonding strength of the γ/α’ Fe interfaces, resulting in the quasi-cleavage fracture (Figure 6) and drastic drop in material ductility (Figure 4 and Figure 5). The isothermal aging treatment was also able to increase the concentration of trapped hydrogen (Figure 7), giving rise to the lower HE resistance [31]. Aging performed at 460 °C leads to the formation of strengthening precipitates. The Ni_3_Ti phase (or more generally, Ni_3_X where X = Ti, Mo, V, and W) readily forms on short-term isothermal aging, followed by Fe_2_Mo or Fe_7_Mo_6_ precipitation [1,2,3,4,5,6]. The fine strengthening precipitates not only increased the strength of the alloy due to precipitation hardening but are also suggested to act as additional hydrogen trapping sites [31]. In addition, the increase in austenite fraction by a diffusion-controlled reaction [12], which is favored by the release of Ni into the matrix due to decomposition of Ni_3_X phase into Fe–Mo precipitates, could also play a role in increasing hydrogen concentration and in the deterioration in the HE behavior of the steel through the above-mentioned mechanisms.

Finally, it is worth mentioning that the specimens showed a relative density of 99.8%. Lack-of-fusion pores that mainly originated from spatters were detected. Spatter particles do not allow proper melting of the powder particles and prevent the homogeneous deposition of powder particles. It is expected that irregular-shape pores play a role in reducing the toughness and ductility of metals and the HE resistance of the steel. Indeed, volume defects serve as fracture initiators and enhance the HE susceptibility of martensitic steels [32]. Excess hydrogen accumulates in pores and internal cracks, and once a critical tensile stress is applied hydrogen promotes fracture propagation and contributes to brittle fracture [32]. Thus, the presence of pores is a detrimental factor for HE of maraging steel produced by SLM or casting. That said, it is difficult to compare the HE behavior of the maraging steel produced by SLM and tested in this work with that of maraging steel produced by conventional methods reported in previous research articles, because of differences in H-charging conditions, the testing environment, or chemical composition [28,33,34,35]. Maraging steels produced both by conventional methods [28,33,34,35] and by AM technologies are very susceptible to hydrogen embrittlement phenomenon. In this research, it was shown that although all tested conditions suffered the effects of hydrogen, solution treatment had a beneficial effect on the HE behavior of the steel, whereas artificial aging to peak hardness had a negative effect. The same trend is also confirmed by research on maraging steels produced by conventional processes [28,33].

## 5. Conclusions

Microstructure characterization and mechanical testing were performed on 18-Ni maraging steel bars produced by SLM and subjected to different heat treatment routes and to H-charging. Solution treatment modified the solidification microstructure typical of high alloyed steels processed by SLM. The solidification cells decorated at the boundaries by a solute-rich region, were replaced by a martensitic structure. Microstructure modifications induced by heat treatments resulted in different hydrogen embrittlement behavior of the steel. A lower number of trapped atoms were detected in the H-charged solution treated samples, and higher ductility was shown under tensile tests compared to the H-charged as-built counterparts. The reduced susceptibility to hydrogen of solution treated steel is attributed to the reduction of austenite and dislocation induced by the heat treatment. Isothermal aging treatment increased the strength of uncharged steel but was also responsible for an increase in trapped hydrogen and lower hydrogen embrittlement resistance.

## Figures and Tables

**Table 1:** Chemical composition of the 18-Ni 300 maraging steel powder. The element concentration is expressed in weight percent (wt.%).

Ni	Mo	Co	Ti	Al	Si	Fe
17.6	5.3	9.6	0.7	0.09	0.2	Bal.
